# Experimental Evolution of the TolC-Receptor Phage U136B Functionally Identifies a Tail Fiber Protein Involved in Adsorption through Strong Parallel Adaptation

**DOI:** 10.1128/aem.00079-23

**Published:** 2023-05-16

**Authors:** Alita R. Burmeister, Eddy Tzintzun-Tapia, Carli Roush, Ivan Mangal, Roxanna Barahman, Robert D. Bjornson, Paul E. Turner

**Affiliations:** a Department of Ecology and Evolutionary Biology, Yale University, New Haven, Connecticut, USA; b BEACON Center for the Study of Evolution in Action, East Lansing, Michigan, USA; c Yale Center for Research Computing, New Haven, Connecticut, USA; d Microbiology Program, Yale School of Medicine, New Haven, Connecticut, USA; e Center for Phage Biology and Therapy, Yale University, New Haven, Connecticut, USA; f Department of Biological Sciences, University of Wisconsin Milwaukee, Milwaukee, Wisconsin, USA; Unversidad de los Andes

**Keywords:** antibiotic resistance, bacteria, bacteriophage, evolution, tradeoff

## Abstract

Bacteriophages have received recent attention for their therapeutic potential to treat antibiotic-resistant bacterial infections. One particular idea in phage therapy is to use phages that not only directly kill their bacterial hosts but also rely on particular bacterial receptors, such as proteins involved in virulence or antibiotic resistance. In such cases, the evolution of phage resistance would correspond to the loss of those receptors, an approach termed evolutionary steering. We previously found that during experimental evolution, phage U136B can exert selection pressure on Escherichia coli to lose or modify its receptor, the antibiotic efflux protein TolC, often resulting in reduced antibiotic resistance. However, for TolC-reliant phages like U136B to be used therapeutically, we also need to study their own evolutionary potential. Understanding phage evolution is critical for the development of improved phage therapies as well as the tracking of phage populations during infection. Here, we characterized phage U136B evolution in 10 replicate experimental populations. We quantified phage dynamics that resulted in five surviving phage populations at the end of the 10-day experiment. We found that phages from all five surviving populations had evolved higher rates of adsorption on either ancestral or coevolved E. coli hosts. Using whole-genome and whole-population sequencing, we established that these higher rates of adsorption were associated with parallel molecular evolution in phage tail protein genes. These findings will be useful in future studies to predict how key phage genotypes and phenotypes influence phage efficacy and survival despite the evolution of host resistance.

**IMPORTANCE** Antibiotic resistance is a persistent problem in health care and a factor that may help maintain bacterial diversity in natural environments. Bacteriophages (“phages”) are viruses that specifically infect bacteria. We previously discovered and characterized a phage called U136B, which infects bacteria through TolC. TolC is an antibiotic resistance protein that helps bacteria pump antibiotics out of the cell. Over short timescales, phage U136B can be used to evolutionarily “steer” bacterial populations to lose or modify the TolC protein, sometimes reducing antibiotic resistance. In this study, we investigate whether U136B itself evolves to better infect bacterial cells. We discovered that the phage can readily evolve specific mutations that increase its infection rate. This work will be useful for understanding how phages can be used to treat bacterial infections.

## INTRODUCTION

Antibiotic resistance is a major global health threat. Antibiotics continue to be used in human health and agriculture, and bacterial populations easily evolve antibiotic resistance. This process has been a cause for alarm (1), with long-standing calls to find new antibiotics, improve their use, and design alternative treatment strategies.

One potential alternative or complement to chemical antibiotics is the use of lytic bacteriophages (“phages”) to kill pathogenic bacteria (2, 3) in a general strategy broadly termed phage therapy. Phage therapies may offer some potential advantages over antibiotics, such as their ability to self-amplify and adaptively respond to the evolution of bacterial populations. Phages may also be used in addition to standard-of-care treatments, for example, alongside current antibiotic-based therapies or in new phage-antibiotic combinations (4). However, phage therapy also faces some of the same challenges as antibiotic-based therapy, including the rapid evolution of resistance over short timescales. Together, these factors limit the utility of phages to suppress bacterial population sizes. Therefore, phages must be carefully chosen and studied for both their therapeutic potential, as well as their potential to limit the effects of phage resistance (5).

The concept of “evolutionary steering” has been proposed as a way to use phage selection to prefer certain evolved bacterial phenotypes (2, 6, 7). Evolutionary steering exploits the idea of the evolutionary trade-off, whereby the acquisition of one beneficial trait (for example, phage resistance) comes with the loss of another beneficial trait (such as pathogenesis). Evolutionary steering in phage therapy works 2-fold: first, through the reduction of bacterial densities directly through phage lysis, and second, through the shift in the bacterial population to a less-pathogenic phenotype. Such shifts may be possible through the use of phages that target various undesirable bacterial features, including those that confer the ability to infect mammalian cells, evade the immune system, form biofilms, or resist antibiotics. When bacteria face selection by such phages, they may evolve phage resistance at the expense of these traits, thereby pleiotropically losing virulence in the process (6).

We previously discovered and characterized one such phage with a well-linked mechanism between phage infection ability and antibiotic resistance. Escherichia coli phage U136B is tightly reliant on the antibiotic efflux pump protein TolC (8). TolC is an alpha-beta barrel outer membrane protein that effluxes a variety of antibiotics as part of various efflux pump systems (9, 10). Phage U136B strictly requires the presence of TolC to infect E. coli: the phage has no growth on *tolC* knockout bacteria, and growth can be rescued by *tolC* expression from a plasmid (8). The phage is obligately lytic (virulent), contains no predicted virulence factors (11), has a genome size of 49.2 kb, and is related to the only other two known TolC-dependent *E. coli* phages (phage TLS and phage LL5), all within the family *Drexlerviridae* (12, 13). Phage U136B has a curly-tailed siphophage morphology and was originally isolated from a swine farm via enrichment of E. coli (8). We have also shown that phage U136B’s reliance on TolC makes it amenable to evolutionary steering, such that populations of E. coli tend to evolve phage resistance at the expense of antibiotic resistance via specific *tolC* mutations (8).

**FIG 1:**
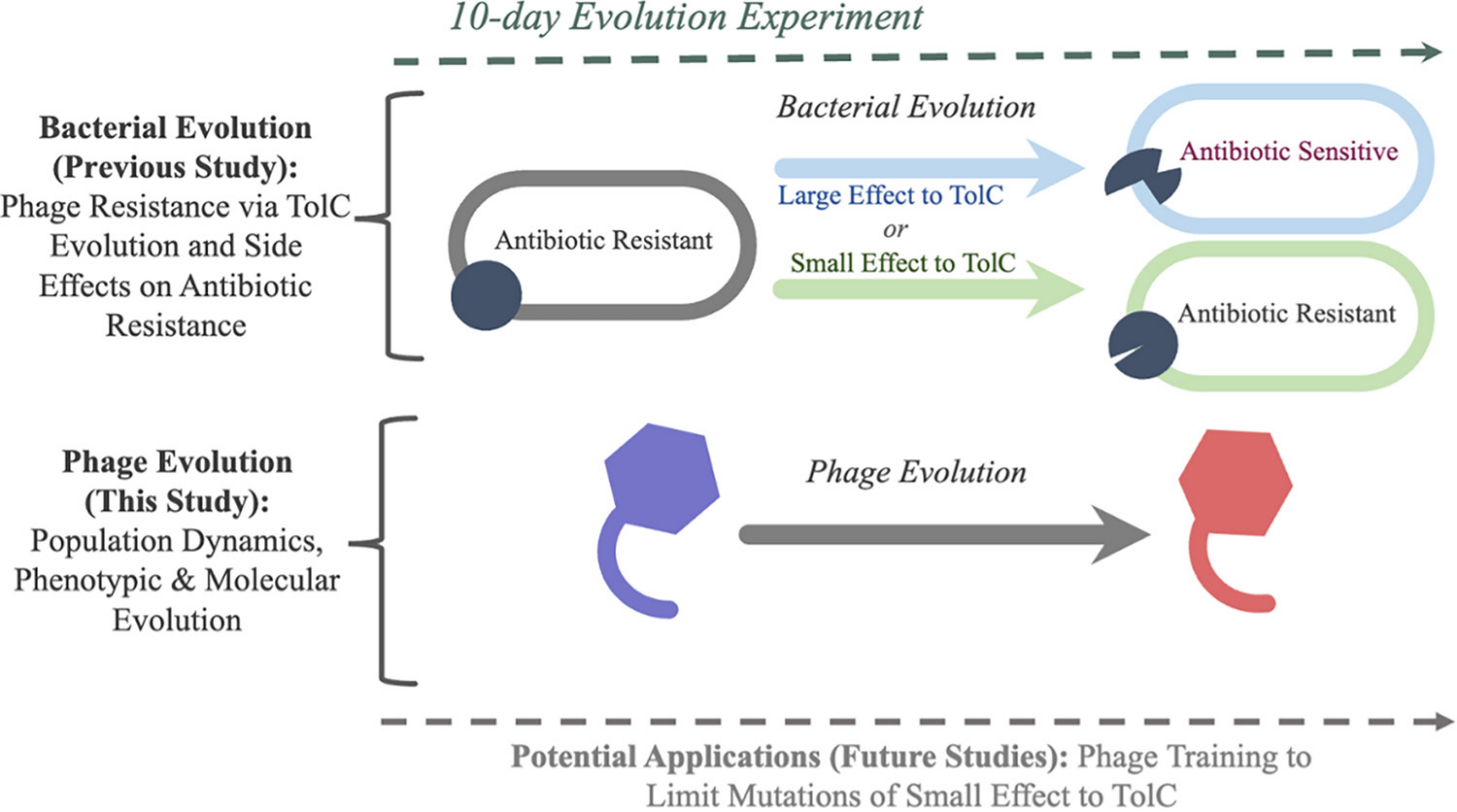
Overview of the study system. During a 10-day evolution experiment, ancestral E. coli evolve under selection pressure by phage U136B. E. coli populations evolve phage resistance through either large-effect changes to TolC (blue, loss-of-function mutations that result in antibiotic sensitivity) or small-effect change to TolC (green, no loss-of-function). Meanwhile, the phage U136B populations also evolve new genotypes and phenotypes, investigated in this study.

If evolutionary steering is to be applied using a phage like U136B, then it is important to also understand how the phages themselves evolve once administered (5). If phages become better at generally infecting their bacterial hosts, these evolved phages may also be useful as therapies to target ancestral or phage-resistant bacteria (14, 15). This approach would be especially useful for generating phages that infect phage-resistant bacterial mutants. For example, phage U136B and the related TolC-reliant phage TLS can sometimes select for bacteria that contain mutations in the target TolC protein but that retain antibiotic efflux function (8, 12, 16). Using evolution to obtain new phage variants that can target those mutant TolC proteins could help slow or prevent host evolution that evades both phage resistance and antibiotic resistance (14) [Fig. 1, Potential Applications (Future Studies)].

During host-phage coculture, host resistance often arises rapidly, commonly through changes to the cell surface that limit phage attachment, and phages often evolve to increase their adsorption rate to host cells. Such evolved changes to the bacteria include direct alterations to the phage receptor gene (for example, through amino acid sequence substitutions, deletions, or insertion sequence disruptions) (8), reductions in expression of receptors (17), or changes to the general property of the cell surface (for example, by increasing the amount of exopolysaccharide coating the cell, which blocks phage access to the surface) (18). The type and level of host resistance can influence whether and how phages can coadapt and improve infectivity (and thereby fitness) on the resistant hosts. The evolution of increased adsorption commonly occurs through mutations in genes encoding host recognition structures, including phage tail proteins (17, 19, 20). However, increased phage adsorption via the evolution of host recognition is not inevitable (21–23). In other cases, mutations are observed in predicted host attachment proteins, but adsorption rate data are unavailable, so the relationship between genotype and phenotype is unknown (24).

Here, we investigate phage U136B’s evolution in terms of phage population dynamics, phenotypic evolution, and molecular evolution (Fig. 1). We find that phage adsorption rapidly evolves during experimental coculture with E. coli. These changes occur by highly parallel evolution (the repeated, independent evolution of similar genotypes/traits from a common ancestor) of phage tail fiber proteins, allowing an evolution-based functional annotation of a phage U136B tail fiber protein and insight into the mode of evolution between E. coli and phage U136B.

## RESULTS

### Host and phage population dynamics.

We previously conducted an evolution experiment with serial passaging of replicate communities of E. coli K-12 strain BW25113 (Table 1) with bacteriophage U136B (Table 1) for 10 days (8). The experiment involved 10 control populations without phage and 10 populations of bacteria + phage (Pop+1 through Pop+10), and samples of all populations were frozen daily. To better understand the ecological and evolutionary dynamics during the experiment, in this study we first characterized the timing and frequency of resistance to the ancestral phage and the density of phages during experimental evolution. The phage density was determined by titrating plaques on a lawn of the parental E. coli BW25113 strain, and we determined the fraction of resistant cells by cross-streaking subsets of community-derived clones with the parental phage U136B.

**TABLE 1:** Bacteria and phages used in this study

Strain	Description (citation)
Bacteria	
BW25113	E. coli K-12, a common laboratory strain and parent of the Keio knockout collection; lacks O-antigen and was derived from ancestral K-12 strain EMG2 (26)
JW5503-1	Δ*tolC732:kan* (40)
AB229	Δ*tolC732:kan* pCA24N::*tolC,* plasmid from reference 41
AB279	Evolved from BW25113, Pop+2 isolate, day 10. Contains a *tolC* mutation resulting in an amino acid substitution of tyrosine to aspartic acid at position 283 (8)
Phages	
U136B	Environmental isolate from a swine farm (8, 11)
ET003	Evolved U136B mutant, Pop+1 isolate, day 5
ET004	Evolved U136B mutant, Pop+2 isolate, day 5
ET005	Evolved U136B mutant, Pop+3 isolate, day 5
ET006	Evolved U136B mutant, Pop+4 isolate, day 5
ET007	Evolved U136B mutant, Pop+5 isolate, day 5
ET008	Evolved U136B mutant, Pop+6 isolate, day 5
ET009	Evolved U136B mutant, Pop+7 isolate, day 5
ET010	Evolved U136B mutant, Pop+8 isolate, day 5
ET011	Evolved U136B mutant, Pop+9 isolate, day 5
ET012	Evolved U136B mutant, Pop+10 isolate, day 5
RB-020	Evolved U136B mutant, Pop+2 isolate, day 10
RB-022	Evolved U136B mutant, Pop+4 isolate, day 10
RB-024	Evolved U136B mutant, Pop+6 isolate, day 10
RB-028	Evolved U136B mutant, Pop+8 isolate, day 10
ET013	Evolved U136B mutant, Pop+9 isolate, day 10

**FIG 2:**
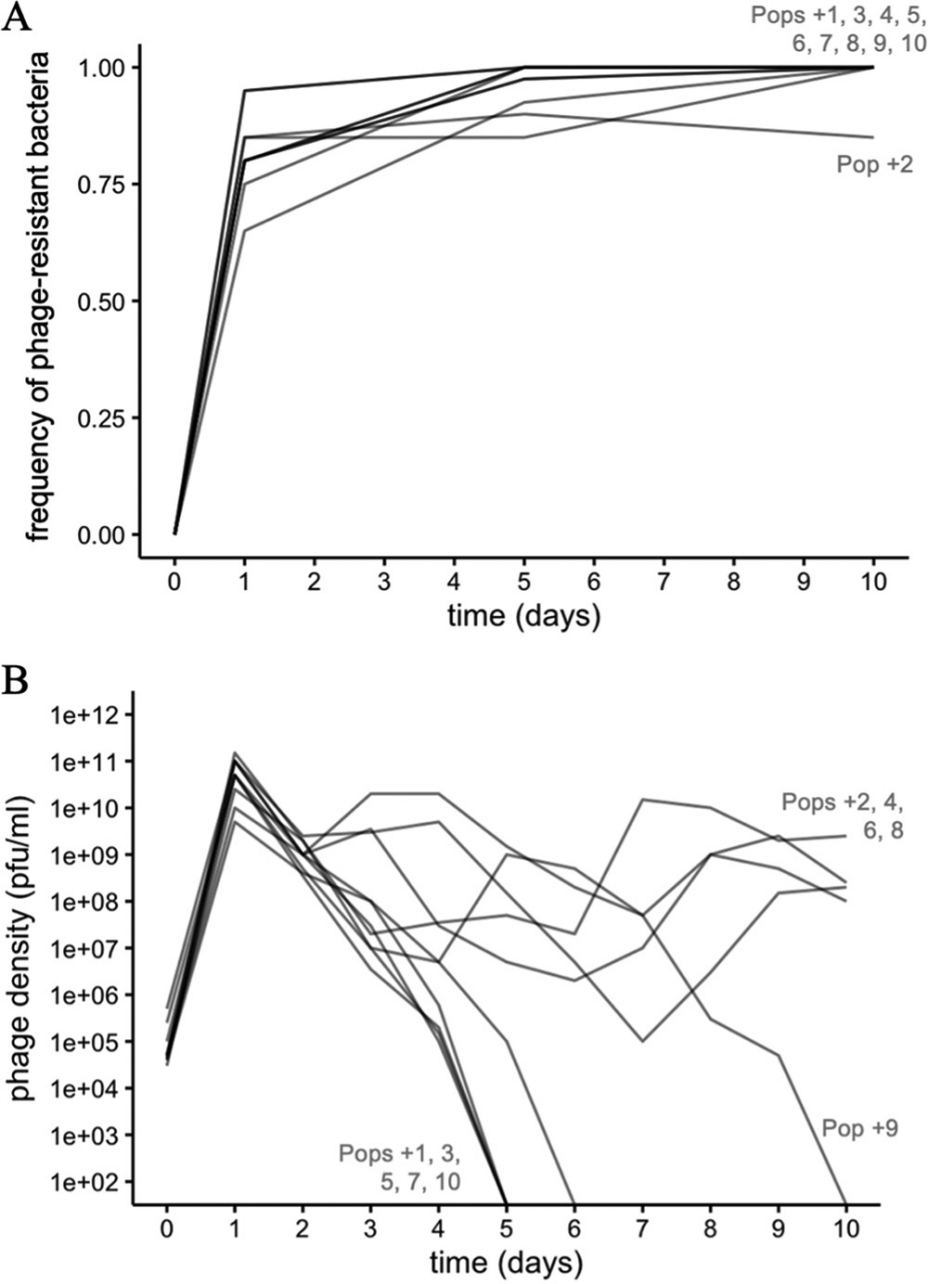
Dynamics during the 10-day evolution experiment. (A) Host bacteria rapidly evolve high levels of resistance to phage U136B within 1 day of the experiment. By day 10, most populations have undetectable levels of phage susceptibility. Each time point represents the testing of 20 (days 1 and 10) or 40 (day 5) bacterial isolates per population for a total of 800 tests across the experiment. The limit of detection for sensitive bacteria is 95% (days 1 and 10) and 97.5% (say 5). (B) Phage population dynamics. Phage densities were measured on each day during the evolution experiment, with a limit of detection of approximately 100 PFU/mL on the ancestral bacteria. Lines represent the 10 independently evolved populations.

Resistance to phage U136B rapidly evolved in all 10+phage populations (Fig. 2A), becoming the majority phenotype in the population after 1 day and continuing to increase in most populations through 10 days. In 9/10 populations on day 10, we observed complete phage resistance (phage susceptibility dropped below our 5% limit of detection), suggesting resistance reached evolutionary fixation (25).

All 10 phage population densities rapidly increased for 1 day, indicating expected growth on the initial phage-sensitive bacteria, and then the 10 populations diverge (Fig. 2B). Five of the phage populations declined to extinction around day 5 (“extinction-bound populations”). The other five populations persisted until the end of the experiment (“survival-bound populations”). One population, Pop+9, fell below the limit of detection on day 10. However, we were able to retrieve a few isolated phages at very low densities (below the limit of detection with our standard enumeration methods) in this population for phenotypic characterization. Together, these results reveal that phage populations can either persist or go extinct.

### Evolution of plaque size.

**FIG 3:**
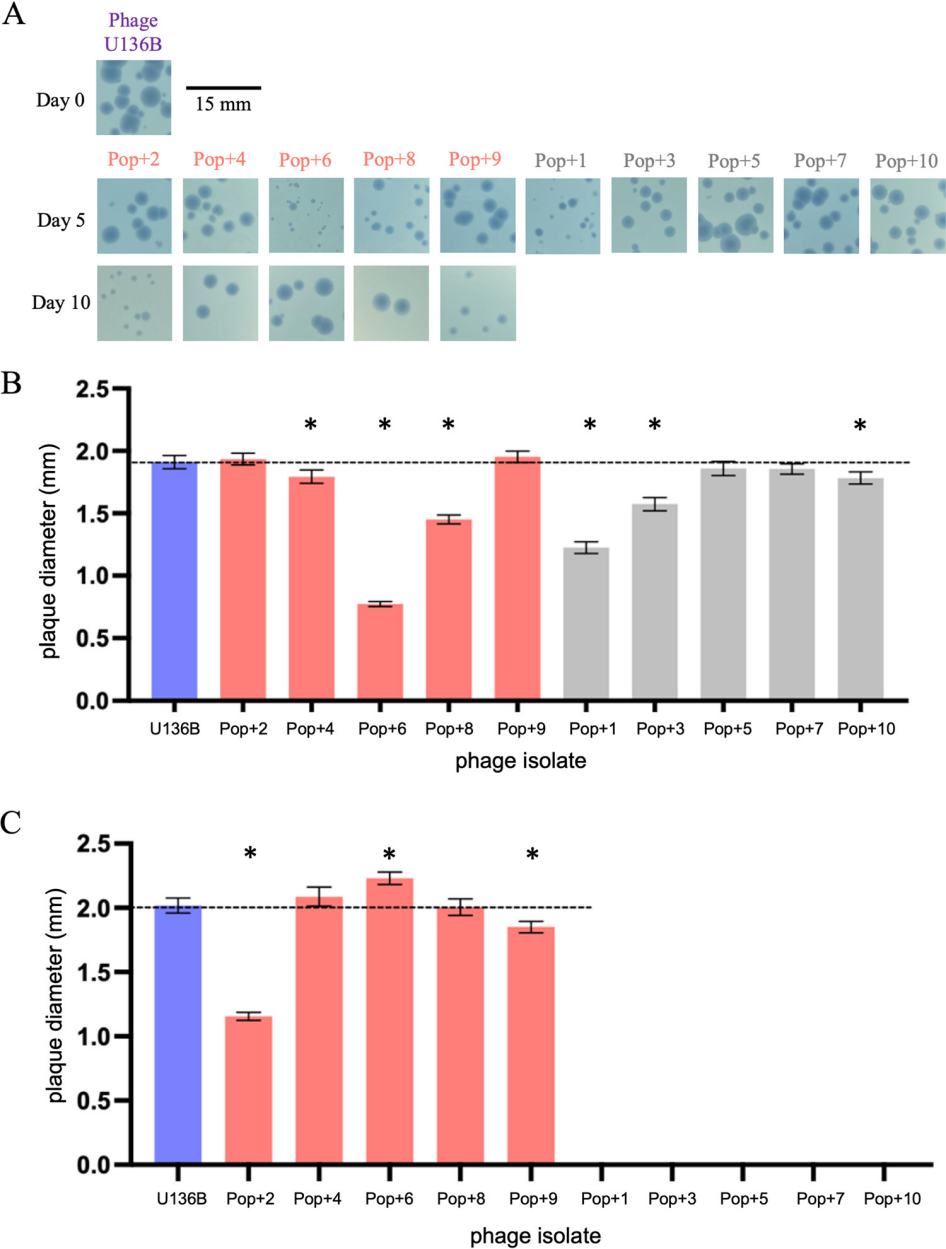
Evolution of phage U136B plaque size during serial passaging with E. coli. (A) Evolved phage isolates were obtained from each of 10 evolved host-phage communities and plated on ancestral bacteria, revealing variation in plaque size among populations. Representative plaques are shown in the photos, and hundreds of plaques (range 357 to 1354) were analyzed using Image J for the quantitative results in panels B and C. (B) On day 5, six evolved phage isolates had significantly reduced plaque sizes compared to the ancestral U136B phage (Pop+4, Pop+6, Pop+8, Pop+1, Pop+3, and Pop+10). Pink bars indicate survival-bound populations and gray bars indicate extinction-bound populations. Error bars indicate 95% CIs, and asterisks indicate significantly different means based on unpaired two-tailed *t* tests between each isolate and the ancestral phage U136B after Holm-Bonferroni correction (*P* values are listed in the main text). (C) On day 10, plaque size was significantly smaller for isolates from Pop+2 and Pop+9 and significantly larger for Pop+6. Phage isolate details are listed in Table 1. Statistical analysis was performed and reported as in panel B.

During the experiment to quantify phage densities (Fig. 2), we informally noticed that the evolved phage plaques varied in size, appearing to become smaller over time (Fig. 3A). To quantify these changes, we developed a photo analysis technique using ImageJ that could measure hundreds of plaque sizes in parallel. Using this method, we measured the plaque size of evolved phage isolates grown on lawns of the ancestral host, BW25113 (Table 1). To do this, we randomly isolated phage clones from frozen samples of each of the 10 populations on day 5 and each of the five surviving populations on day 10 (Table 1). On day 5, when all phage populations remained extant (Fig. 3B), measurements confirmed our initial observation that plaque sizes were reduced for some but not all evolved isolates. Phage isolates from six populations had significantly smaller plaque diameter than the ancestor (Pop+4, *P* = 0.0026; Pop+6, *P* < 0.0001; Pop+8, *P* < 0.0001; Pop+1, *P* < 0.0001, Pop+3, *P* < 0.0001; and Pop+10, *P* < 0.0001, two-tailed *t* tests and significance reported after Holm-Bonferroni correction for multiple comparisons), and no phages had significantly increased plaque diameter. On day 10, two evolved phage isolates had significantly decreased plaque size compared to wild type (Pop+2, *P* < 0.0001; Pop+9, *P* < 0.0001) and one had significantly increased plaque size (Pop+6, *P* < 0.0001) (Fig. 3C).

### Phage U136B adsorption to *tolC*.

**FIG 4:**
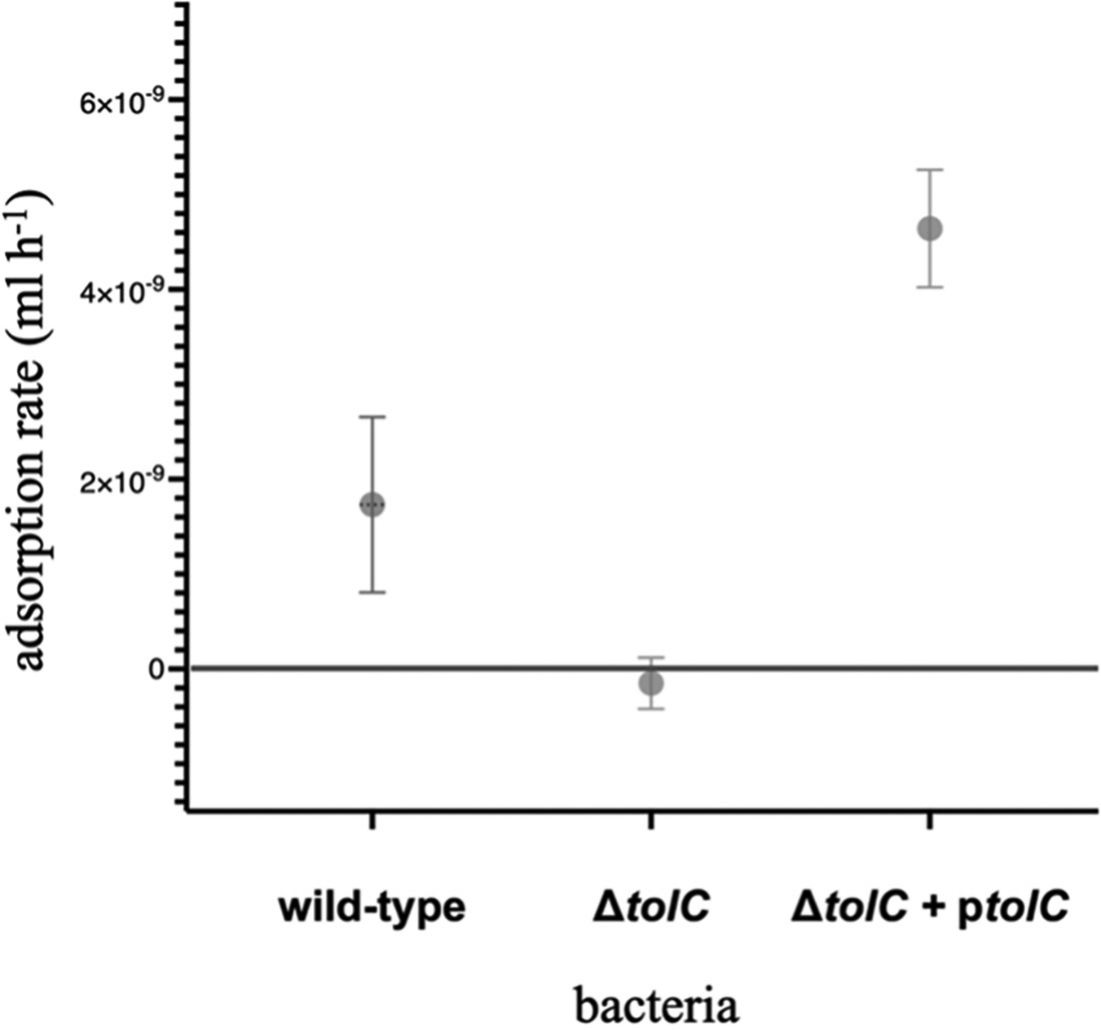
Phage U136B requires TolC for adsorption. Phage U136B adsorbs to wild-type cells (BW25113) but not the Δ*tolC* knockout (AB229). Expression of *tolC* from a vector (Δ*tolC* + p*tolC*, JW5503-1) restores adsorption. Error bars denote 95% confidence intervals. Strain details are given in Table 1.

To test if the evolved phages had increased adsorption rates to TolC, we developed a U136B adsorption assay (see Materials and Methods for details and protocol optimization). Our final protocol confirmed adsorption of phage U136B to wild-type (BW25113) cells but not *tolC* knockout cells (Fig. 4). Expression of *tolC* from a vector (p*tolC*) rescues growth in *tolC* knockout cells (Fig. 4). Adsorption rate is greater on the plasmid containing strain compared to wild type, which we attribute to TolC overexpression via an IPTG-inducible promoter. Overall, these results confirm that U136B is dependent on TolC for infection in general, and they show specifically that TolC is needed for phage adsorption.

### Evolution of adsorption rates.

**FIG 5:**
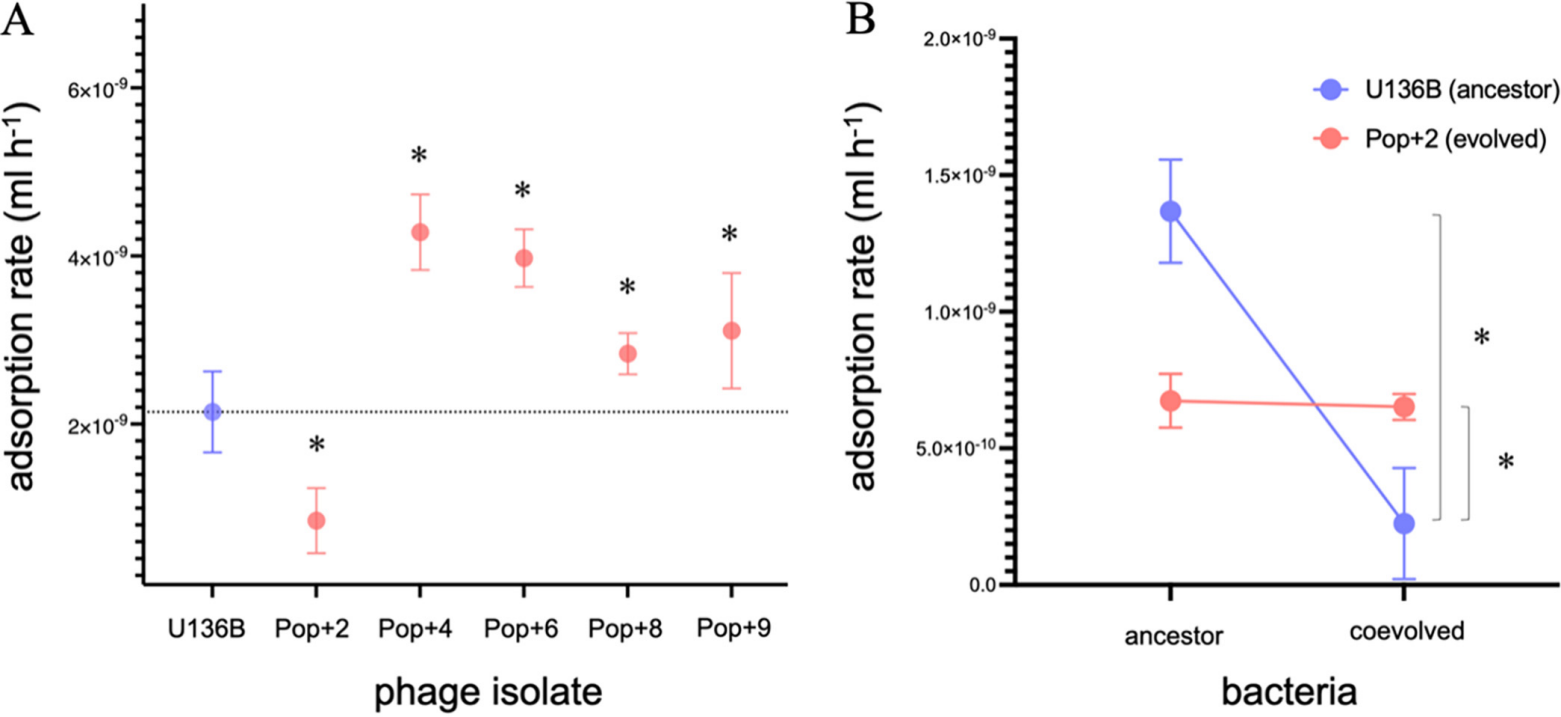
Phage U136B evolves changes in adsorption rates. (A) Phage isolates from Pop+4, Pop+6, Pop+8, and Pop+9 have increased adsorption rate on the ancestral host (BW25113). Phage from Pop+2 has decreased adsorption rate to the ancestral host. The dotted line indicates the mean adsorption rate of the ancestral U136B; *n* = 9 for each test. Error bars indicate 95% CIs, and asterisks indicate significantly different means based on unpaired two-tailed *t* tests between each isolate and the ancestral phage U136B (Pop+2, *P* = 0.0002; Pop+4, *P* < 0.0001; Pop+6, *P* < 0.0001; Pop+8, *P* = 0.0121; and Pop+9, *P* = 0.0173, significance reported after Holm-Bonferroni correction). (B) The phage isolate from Pop+2 has increased adsorption on its *coevolved* host (AB279, Table 1) compared to the ancestral phage isolate (*P* = 0.0185, single-tailed *t* test), indicating host-phage coevolution with local adaptation. The ancestral phage has slower adsorption to the coevolved bacteria compared to the ancestral bacteria (*P* = 0.0005, single-tailed *t* test).

We next tested whether the evolved phage isolates had increased adsorption rates. First, we tested the phage isolates from the five surviving populations on day 10 (the same isolates shown in Fig. 3) for adsorption to the ancestral bacteria BW25113. Four of the five phages had significantly increased adsorption rates compared to the ancestor phage (Fig. 5A).

The fifth-phage isolate (Pop+2) was unique in that it had a significantly *decreased* adsorption rate compared to the ancestral host. We hypothesized that this loss of adsorption rate reflected the local adaptation of the phage population to its own coevolving host. Coevolving hosts would be at higher abundances than the ancestral phage type throughout most of the 10 days of serial growth, and so selection would favor phages that could infect these over the ancestral host. To test this idea, we compared the adsorption of the Pop+2 phage isolate to both the ancestral host BW25113 and a coevolved bacterial clone from day 10 of the Pop+2 community (AB279, Table 1). As we predicted, the evolved Pop+2 phage isolate had a significantly increased adsorption rate on the coevolved bacteria (Fig. 5B). The Pop+2 phage isolate also had slower adsorption to the ancestor compared to the coevolved cells, suggesting a trade-off in terms of host infectivity during coevolution: improving adsorption on the coevolved host resulted in worse adsorption to the ancestral host.

We also have observed no evidence that the phage evolves to use any other outer membrane protein receptor, as none of the phage isolates have any plaquing on a *tolC*-knockout host JW5503-1 (efficiency of plating lower than the limit of detection of 10^−7^ compared to plaquing on wild-type BW25113).

### Genome evolution.

To understand the molecular basis of these adaptations, we sequenced the genomes of the individual, randomly selected phage isolates from each of the five surviving populations on day 10 (the same isolates tested for adsorption rate in Fig. 5) and compared the reads to the U136B reference genome (11). To do this, we extracted DNA from the phage isolates, collected whole-genome sequencing data to a high coverage level (Table S1 in the supplemental material), and identified evolved mutations using *breseq* (26).

All five individual isolates showed molecular evolution associated with changes to host recognition, and these changes were highly parallel. Among the 5 genotypes, there were 11 total unique mutations (Table 2) resulting in 9 unique amino acid changes, with an average of 3.4 mutations per isolate. These mutations occurred via extensive parallel evolution at the level of the gene and gene function (Table 2).

**TABLE 2:** Parallel evolution of tail fiber proteins reveals adaptation associated with host recognition in phage U136B^*a*^

						Phage isolate (source population)				
Genome position	BP change	Amino acid change (codon)	Impact on amino acid	Gene no.	Putative gene function	RB-020 (Pop+2)	RB-022 (Pop+4)	RB-024 (Pop+6)	RB-028 (Pop+8)	ET013 (Pop+9)
28,296	C→A	H1186N(CAT→AAT)	Positive to neutral, large to small	52	Tail fiber protein		x			
28,321	C→A	T1194K (ACG→AAG)	Neutral to positive, small to large	52	Tail fiber protein		x			
*28,380	C→A	Q1214K (CAA→AAA)	Neutral to positive and small to large	52	Tail fiber protein		x		x	x
28,381	A→G	Q1214R (CAA→CGA)	Neutral to positive and small to large	52	Tail fiber protein	x				
28,383	G→A	A1215T (GCT→ACT)	Nonpolar to polar	52	Tail fiber protein				x	
28,411	G→T	G1224V (GGA→GTA)	Polar to nonpolar	52	Tail fiber protein					x
28,463	C→G	N1241K (AAC→AAG)	Neutral to positive, small to large	52	Tail fiber protein	x		x		
28,469	C→A	N1243K(AAC→AAA)	Neutral to positive, small to large	52	Tail fiber protein	x				X
31,917	C→A	K688N (AAG→AAT)	Positive to neutral, small to large	57	Tail fiber protein			x	x	X
35,091	(A)_8→7_	None, intergenic	Not applicable	58–59	DNA primase, transcrip. regulator		x			
38,215	(T)_5→4_	None, intergenic	Not applicable	61–62	Endonuclease, hyp. protein		x			

a Mutations were identified by whole-genome sequencing and comparison to the U136B reference genome (GenBank no. MW598258.1). Genome position column indicates location in the genome in bp. BP change column indicates the nucleotide mutation. Amino acid change (codon) column indicates amino acid change and codon change using standard genetic code abbreviations (e.g., H1186N indicates a change from histidine to asparagine at amino acid position 1186, corresponding to a change from cytosine to adenine in the first position of the codon). Impact on amino acid column indicates changes to the amino acid properties. Gene no. column indicates the gene product number within the U136B genome. Putative gene function column indicates the bioinformatic-based manual gene annotation as previously reported (11). Asterisk indicates mutation was detected in original phage stock; see description in the main text. Light gray shading and an “X” indicates the mutation was present in the indicated population.

All five phage isolates (100%) had nonsynonymous (resulting in an amino acid change) mutations in a putative tail fiber protein (gene 52, with an open reading frame length of 3,776 bp), and three (60%) had nonsynonymous mutations in another putative tail fiber protein (gene 57, with an open reading frame length of 3,372 bp). It is highly unlikely that these mutations arose by chance alone through nonselective processes. As a very conservative estimate, the probability of any mutation arising in all five populations in gene 52 can be calculated as *P* = (length_gene_/length_genome_)^4^ = (3,776/49,223)^4^ = 0.00003. The probability for a mutation in three populations in gene 57 is correspondingly *P* = (2,372/49,223)^2^ = 0.002. Together, the genomic mutations of the phage isolates reveal strong selection in genes with previously predicted (putative) host recognition function.

To determine if the individual phage isolate genomes (Table 2) were representative of the evolved populations they were sampled from, we also analyzed DNA sequence reads from each of the entire phage populations on day 10 (Table 3). To do this, we extracted DNA from samples of each entire community of bacteria and phage, sequenced the entire community’s DNA pool, and aligned the mixed reads to the ancestral phage genome using *breseq* in polymorphism mode, which can detect variation in a population (see Materials and Methods). We detected phage sequences in 4/5 surviving populations (all except Pop+9, where phages existed in very low densities at the end of the experiment; Fig. 2A). Four of four populations had multiple nonsynonymous mutations in tail fiber protein 52, tail fiber protein 57, or both (Table 3). Multiple populations also contained lower frequencies of nonsynonymous mutations in genes not found in our individual isolates, including a putative portal protein (involved in DNA packaging and connects head and tail components, gene 33), a putative tail tape measure protein (the structural component that determines the length of the tail, genes 46 and 47, which share an open reading frame via a ribosomal slipper sequence), a methylase (gene 63), and a hypothetical protein of unknown function (gene 85). The occurrence of these alleles in multiple populations suggests that they could be adaptive and may be either in the process of sweeping through the population or maintained by negative frequency-dependent selection.

**TABLE 3:** Genomic evolution within U136B populations^*a*^

						Phage population			
Genome position	BP change	Amino acid change (codon)	Impact on amino acid	Gene no.	Putative gene function	Pop+2	Pop+4	Pop+6	Pop+8
11,048	T→G	R116R (CGT→CGG)	Synonymous	33	Portal protein	6.7%	7.6%	6.9%	
19,245	T→G	None, Intergenic	Not applicable	046–047	TMP-TMP protein		9.5%		
19,261	A→T	V3V (GTA→GTT)	Synonymous	47	Tail tape measure protein	5.6%	5.7%	5.4%	
19,272	C→G	A7G (GCT→GGT)	Nonpolar to polar	47	Tail tape measure protein		5.0%		
28,296	C→A	H1186N (CAT→AAT)	Positive to neutral, large to small	52	Tail fiber protein		100%		
28,321	C→A	T1194K (ACG→AAG)	Neutral to positive, small to large	52	Tail fiber protein		100%		
*28,380	C→A	Q1214K (CAA→AAA)	Neutral to positive, small to large	52	Tail fiber protein		100%	100%	100%
28,381	A→G	Q1214R (CAA→CGA)	Neutral to positive, small to large	52	Tail fiber protein	100%			
28,383	G→A	A1215T (GCT→ACT)	Nonpolar to polar	52	Tail fiber protein				100%
28,446	C→A	L1236I (CTT→ATT)	Nonsynonymous conserved	52	Tail fiber protein			100%	
28,463	C→G	N1241K (AAC→AAG)	Neutral to positive, small to large	52	Tail fiber protein	100%		100%	
28,469	C→A	N1243K (AAC→AAA)	Neutral to positive, small to large	52	Tail fiber protein	100%			
31,700	+82 bp	+82 bp (2281/2373 nt)	Frame-shifting insertion	57	Tail fiber protein				77.2%
31,718	+86 bp	+86 bp (2263/2373 nt)	Frame-shifting insertion	57	Tail fiber protein			63.6%	
31,917	C→A	K688N (AAG→AAT)	Positive to neutral, small to large	57	Tail fiber protein			100%	100%
38,211	Δ1 bp	none, intergenic	Not applicable	061–062	Endonuclease, hypothetical protein		100%	
39,132	T→G	F192V (TTT→GTT)	Large to small	63	Methylase		5.0%	5.3%	
39,139	C→A	S194Y (TCT→TAT)	Polar to nonpolar, small to large	63	Methylase		6.6%	6.9%	
48,412	G→T	S15* (TCA→TAA)	Early stop codon	85	Hypothetical protein	5.8%	5.1%	5.4%	
48,438	G→T	F6L (TTC→TTA)	Large to small	85	Hypothetical protein	8.9%	8.6%	8.9%	
48,444	T→A	L4F (TTA→TTT)	Small to large	85	Hypothetical protein	6.4%	5.5%	5.5%	
48,446	A→C	L4V (TTA→GTA)	Nonsynonymous conserved	85	Hypothetical protein	10.6%	9.9%		

a Percentages indicate the frequency of DNA sequencing reads supporting each mutation in each population, with “100%” (dark gray shading) indicating that no other alleles at that locus were detected, and other percentages (light gray shading) indicating multiple alleles were detected. For populations with phage extinctions (Pop+1, Pop+3, Pop+5, Pop+7, and Pop+10) or near extinction (Pop+9), no phage DNA was detected. Genome position column indicates location in the genome in bp. BP change column indicates the nucleotide mutation. Amino acid change (codon) column indicates amino acid change and codon change using standard genetic code abbreviations (e.g., H1186N indicates a change from histidine to asparagine at amino acid position 1186, corresponding to a change from cytosine to adenine). Impact on amino acid column indicates changes to the amino acid properties. Gene no. column indicates the gene product number within the U136B genome. Putative gene function column indicates the bioinformatic-based manual gene annotation as previously reported (11). Asterisk indicates mutation was detected in original phage stock; see main text.

Comparing the sequence results obtained for our isolates to those from the whole population revealed that the two data sets are generally consistent, in particular for the general parallel evolution of the tail fiber proteins. We also observed three cases of mutations that show up in 100% of the population-level DNA sequences but did not appear in the individual isolates. Specifically, the Pop+6 isolate (RB-024, Table 2) does not contain mutations Q1214K and L1236I in phage tail protein 052, which showed up in 100% of population reads (Table 3), and the Pop+4 isolate RB-022 (Table 2) does not have a 1-bp deletion between a putative endonuclease (gene 061) and hypothetical protein (gene 062). These differences could arise from sampling effects or from methodological differences between the isolation of phage plaques versus whole population DNA extractions, but they highlight the complementarity of isolate sequencing and whole population sequencing for measuring genetic evolution. These mismatches show that it is important to not overly interpret DNA read frequencies with actual mutant frequencies within evolving populations and that isolating and sequencing individual isolates, as we have done here, can give a fuller picture of population diversity and phage adaptations.

Although we started each of the 10 phage evolutionary lineages independently (each grown from a separate plaque and thus each originating with minimal within-population genetic variation), it is possible that if mutations in the original phage stock were at a high enough density, then they might have been preexisting in more than one of the lineages. To check if any of the evolved mutations may have been present at the onset of the experiment, we also ran *breseq* on DNA reads collected from ancestral lab stock of phage U136B. We reached an average sequencing coverage of 663× and found evidence of the following two polymorphisms in this stock:

(i) Mutation Q1214K in gene 52, which appeared in 30.4% of reads in the ancestral stock and in three of the day 10 evolved isolates (Pop+4, Pop+8, and Pop+9, Table 2). This result indicates that this particular mutation likely existed from the time of inoculation in these populations, and so we do not consider it to have evolved in parallel (Conceptual Figure S1).
(ii) A duplication of 86 bp at the 31,648-bp position (within gene 57) in 15.9% of reads in the ancestral stock. We did not observe this mutation in any of our evolved isolates, so it is unlikely that it existed from the time of inoculation or impacts our results on parallel evolution.

## DISCUSSION

Using adsorption assays and genetic complementation, we confirm TolC receptor use for phage U136B and show that this phage rapidly evolves changes to host adsorption rates. Combining these phenotypic data with whole-genome sequencing, we show that previously putative phage tail fiber genes are directly involved in host adsorption. This work will be useful for future studies aimed at developing TolC-specific phages for therapeutic use, including evolutionary steering, resistance control, and phage training.

Parallel evolution, the repeated, independent evolution of similar genotypes and phenotypes, is a sign of adaptation, so it is useful in understanding the major selection pressures acting on phages. From an applied perspective, parallel evolution could also be useful if evolution unfolds in a predictable way. If phage populations generally evolve the same traits, then it may be less likely that evolution could take unexpected or undesirable turns while being used therapeutically, for example, by phages evolving to target commensal bacteria (27). In the most repeatable cases, parallel evolution of phages occurs not only in the same genes, but in the same specific protein domains, sometimes even affecting just a few amino acids (17). Our results show that phages evolve in parallel in all populations, arriving at similar phenotypes (Fig. 5) and genotypes (Table 2 and 3).

Our genomic sequences also allow us to improve the functional annotation of phage U136B by linking genotype directly to phenotype. Typically, to functionally ascribe a mutation to a gene function in an experimentally evolved isolate, one would need to construct an isogenic strain to remove the effects of cooccurring mutation in other genes. However, one phage (Pop+2) only contained mutations in the putative tail fiber protein 52, with no other changes anywhere else in the genome, either intergenic or intragenic (Table 2), and so this isolate is already isogenic with respect to the evolved putative tail fiber protein 52. Therefore, this tail fiber protein (phage gene product 52) is directly linked to the change in this phage isolate’s increased adsorption rate phenotype. The changes in the other putative tail fiber protein (phage gene product 57) cooccur with other mutations, and so while they are implicated in phage adsorption evolution, further work would be needed to isolate those mutations and determine their direct effects.

Coevolution is a special case of a dual adaptation, often occurring when hosts evolve resistance followed by reciprocal phage counteradaptation. The two major models that are often applied to host-phage coevolution include inverse gene-for-gene (IGFG) and matching alleles (MA) coevolution (19, 28–31). During IGFG dynamics, hosts lose structures important for phage growth, and phages subsequently evolve to exploit new structures, for example, when E. coli evolves resistance to lambda through loss of sugar transporters and lambda evolves to exploit new membrane proteins (17, 32). Conversely, during MA evolution, hosts would modify structures important for phage growth, and the phage would subsequently evolve genotypes that better match the modifications. This appears to partially be the case in one aspect of E. coli-lambda coevolution, which occurs when lambda evolves alleles that allow improved adsorption to a current receptor (19, 33). Using host and phage genomic sequences can be helpful in determining the model (e.g., IGFG versus MA models) and mode (arms race dynamics versus fluctuation selection dynamics) of coevolution, which in turn can be useful for understanding and predicting the ecology and evolution of phages and their hosts (28, 34).

Although understanding the genetic networks and mode of coevolution was not a direct goal of our study, our Pop+2 data hint toward a MA model of coevolution, in which hosts evolved mutations in *tolC* that severely limited phage infection (in this case, a single amino acid substitution, Table 1), and phage countermutations (three amino acid substitutions in tail fiber protein 52) allowed improved infection on the mutant TolC proteins. However, unlike perfect MA models, the Pop+2 phage does retain some infection ability on the ancestral host. Additionally, in the other populations (Pop+4, Pop+6, Pop+8, and Pop+9), phages retained strong infection on the ancestral host, suggesting that the MA model is not a common mode of coevolution among populations in this study system. Instead, phages may be evolving to better infect very rare ancestral-type hosts that persist alongside phage-resistant hosts (35). It may also be possible that phages from other communities were coevolving with their hosts; however, we have been unable to isolate day 10 phages that plaque on day 10 hosts from their same populations. Because of this, we have not been able to investigate the strength of interactions between phage and host genotypes in those populations, as we have for Pop+2 (Fig. 5B). Nevertheless, our results show that all phages evolved faster adsorption rates on either ancestral or evolved hosts (Fig. 5A), and future work will be useful for uncovering rare host-phage interactions in these populations and in determining the model and mode of coevolution.

We additionally observed plaque size evolution (Fig. 3). In another E. coli phage (lambda), plaque size has been associated with phage adsorption rate, with faster adsorbing phage genotypes producing smaller plaques (36). This effect is presumably because they rapidly infect local cells, thereby diminishing their ability to diffuse and create a large zone of lysis. Therefore, we had expected to generally see the evolution of smaller plaques in phage U136B, which was indeed sometimes the case (Fig. 3B and C). In other cases, plaque size was unchanged or even greater when plated on the ancestral bacteria. These results suggest plaque size is a complex phenotype that may depend on a variety of factors, such as the host strain used for plating and interactions among phage mutations. Indeed, we found that plaque size and efficiency were not indicative of phage fitness as measured by adsorption rates (Fig. S2 and Fig. 5B). Future studies aimed specifically at understanding phage growth in top agar overlays will be needed to better understand factors that influence plaque size.

Understanding how TolC-specific phages evolve also has utility for two aspects related to phage applications. First, understanding how phages evolve could be important to predicting how they may change during therapeutic or applied contexts. If phages change significantly due to *de novo* evolution during applied use, this could have implications for what breadth of phenotypes and genotypes are acceptable for drug regulatory purposes. Second, phage evolution *in vitro* and *in vivo* may be useful for isolating phages that can better control bacterial populations (14, 15). Combining preevolved phages into phage mixtures in particular may be useful for “resistance control” strategies that limit the growth of both wild-type and commonly evolved phage-resistant bacteria.

## MATERIALS AND METHODS

### Review of the evolution experiment.

We previously conducted replicate evolution experiments with E. coli strain BW25113 and bacteriophage U136B (8) (Fig. 1). We review some of the details of that experiment here for a convenient reference, in particular with respect to the experimental design needed to make inferences about parallel evolution. In the evolution experiment, we took care to initiate each of the 10 populations from 10 individual phage stocks to avoid the problem of preexisting mutations obscuring results: (i) each of the 10 phage stocks was prepared from an individual plaque and therefore was likely to have arisen from a single individual phage particle (i.e., a single phage genome), minimizing the potential for preexisting variation across replicate populations; (ii) to prepare a phage stock, a single phage plaque was picked off a plate, resuspended in 750 μL of LB and filtered through a 0.2-μm filter without the need for further propagation, thus limited phage growth, and mutation, in the stock; and (iii) to further minimize initial standing genetic variation, we initiated each population with a relatively low density of phages (10^4^ PFU/mL), so that any preexisting mutant would need to reach the fairly high frequency of 10^−4^ during stock preparation. For bacterial evolution, each of the replicates was also initiated from a unique culture grown from a unique colony, and our prior work details the genotypes and phenotypes of evolved bacteria (8).

### Bacterial culture propagation.

Cultures were grown from individual colonies in sterile LB broth (10 g of tryptone, 5 g of yeast extract, and 10 g of NaCl, per 1 L of ultrapure water). Cultures were incubated overnight at 37°C while shaking at 200 rpm.

### Phage population dynamics.

Phage population sizes were monitored daily during the serial passaging experiment. Serial dilutions of the whole community were plated onto the lawn of the parental strain using the conventional double-layer technique. (We find that including the bacteria in the top agar overlays greatly increases observable phage densities, likely because many phages have already adsorbed to or ejected their genomes into host cells.) Plates were incubated at 37°C overnight, and plaques were counted at the dilutions in which individual plaques appeared. Because not all evolved phages may retain the ability to plaque on the ancestral hosts, this approach provides a conservative measure of phage population size.

### Bacterial resistance dynamics.

After coevolution with phage U136B, bacterial isolates from days 1, 5, and 10 from each population were assayed for resistance to the ancestral phage U136B. We used the cross-streak method for determining evolved resistance to phage U136B (8). Bacteria were plated onto LB agar and incubated overnight at 37°C. Individual colonies were randomly picked and streaked across a line of 10 μL of a high-titer bacteriophage stock of U136B using a sterile plastic inoculating loop. For days 1 and 10, we tested 20 bacterial colonies from each time point for each population, and for day 5, we tested 40 bacterial colonies from each time point for each population (total of 800 tests across the experiment). If a clearing was present at the intersection of the two streaks, we marked the bacteria as sensitive.

### Phage plaque size quantification.

We developed a high-throughput protocol to photograph and measure plaque size using ImageJ2 Fiji (version 2.1.0/1.53c). All plaques were obtained through our standard LB top agar overlay method with E. coli strain BW25113 as the host lawn in top agar containing 7.5 g/L agar. For each top agar overlay plate containing plaques, we placed the plate facing upwards over a PetriViewer Model PV-100 with the bright field setting. A smartphone camera (iPhone SE) was attached to a stand perpendicular to the plate viewer 13 cm above the plate. The positioning of the camera and plates for all images was standardized. Images were taken in standard mode, exported to JPEG format, and opened in ImageJ. The diameters of the imaged plates were standardized to 86 mm and this value was used to set the scale from pixels to millimeters. We made a standardized elliptical selection (width of 72.24 mm and height of 72.24 mm) of each plate. The images were then converted to 8-bit grayscale and the “Subtract Background” feature was applied with 50.0 pixels to correct for uneven background lighting. Thresholds of 180 (lower) and 238 (upper) were then applied to maximize the number of plaques analyzed. Finally, the “Analyze Particles” function was used to identify plaques, followed by selection for particles with areas between 0.2 and 64 mm^2^ and with a circularity parameter between 0.20 and 1.00 units. We found that these values helped minimize background noise and lawn aberrations from being mistakenly counted as plaques. Plaque size distributions were comparable to those obtained manually with a millimeter-scale ruler (data not shown).

### Double isolation of evolved phage mutants.

Phage clones were double-isolated through plaque purification from frozen mixed populations of the experimentally evolved communities. First, we used a sterile pipette tip to scrape a small amount of from a −80°C frozen cryo (typically a few μL) and patched it onto the center of an LB agar plate. We then poured top-agar overlays with 100 μL of host bacteria added to 4 mL of 7.5 g/L LB molten (50°C) top agar and gently swirled the plate to distribute plaque-forming units. (We find this method reduces the number of plates needed to obtain plaques and prevents unnecessary freeze-thaw cycles of our cryo vials.) After overnight incubation at 37°C, isolated plaques were picked at random and plated in the same manner with a new top agar overlay and incubated overnight at 37°C. An individual plaque was picked from the second plate at random and suspended in 750 μL of LB broth in a 0.22 μM Spin-X centrifugation filter tube (Corning no. 8160). The tube was centrifuged for 1 min at 14,000 rpm, and 100 μL of the supernatant was transferred to a flask containing 100 μL of host bacteria and 9.8 mL of LB broth. The flask was incubated overnight at 37°C with shaking at 200 rpm. The following day, the contents of the flask were transferred to a 15-mL Falcon tube and centrifuged at 4,000 rpm for 15 min. The supernatant was then filtered through a 0.22-μm filter and stored at 4°C.

### Adsorption assay.

Host cultures were grown overnight in LB broth at 37°C to their final stationary-phase density of 2 × 10^9^ CFU/mL. For the experiment using +pTolC bacterial hosts, overnight cultures were prepared in LB with isopropyl β-d-1-thiogalactopyranoside (IPTG) to a concentration of 100 μM (to induce *tolC* expression from the IPTG-inducible promoter) and chloramphenicol to a concentration 30 μg/mL (to maintain the plasmid). Each culture was centrifuged at 4,000 rpm for 15 min and the supernatant was removed. The pelleted bacteria were resuspended in LB broth warmed for 30 min at 37°C and prepared with a chloramphenicol concentration of 15 μg/mL to halt bacterial growth and thereby also prevent phage from growing. Then, 990 μL of resuspended host bacteria were added to 1.5-mL tubes followed by 10 μL of phage volume to a final assay concentration of 1 × 10^6^ PFU/mL. The tubes were immediately placed in a shaking incubator at 37°C and 200 rpm for 30 min. Afterward, a 100-μL sample was taken from each incubated tube and diluted in 1.5-mL tubes containing 900 μL of 0.85% sterile saline. Then, 20 μL of chloroform were added to each sample and centrifuged for 1 min at 14,000 rpm. Supernatant samples were further diluted in sterile saline for final phage enumeration. To determine the starting concentration of phages, the above steps were also carried out simultaneously without the addition of host bacteria and phages immediately enumerated. To calculate the phage adsorption rate, we quantified the starting and final concentrations of phage from the diluted experimental samples. One-hundred microliters of each diluted sample was added to tubes containing 100 μL of host bacteria and 4 mL of 7.5 g/L LB top agar, tempered to 50°C, and vortexed. The top agar was poured onto 20 mL LB agar plates and incubated overnight at 37°C. Plaque counts were obtained to determine starting (*T*_0_) and final (*T*_1_) phage concentrations in PFU/mL. Phage adsorption rate was calculated as:

$$-\mathrm{Ln}(\frac{T_{1}\;\mathrm{PFU}/\mathrm{mL}}{T_{0}\;\mathrm{PFU}/\mathrm{mL}})/(\mathrm{Time}\;\mathrm{in}\;\mathrm{hours}\;\times \;\mathrm{bacterial}\;\mathrm{density})$$

### Previous attempts at development of an adsorption protocol.

We report earlier methods here in case they are useful to others trying to optimize protocols for similar phages, especially those that may require overexpression of TolC, for which we encountered several methodological issues. Initially, we analyzed single-step growth curve data to directly compare the adsorption of U136B to wild-type and Δ*tolC* cells, with no reliably detectable adsorption in this environment (LB broth) for either cell type (8). Next, to amplify the signal of adsorption, we tried two adsorption assays from the literature that rely on heat-killing the cells and using a longer assay length. We tried the heat-killing method to gently inactivate the cells (20) but found that after treatment, our bacterial cultures formed a clumpy precipitate. Next, we used the shorter, lower-temperature heat-killing method of Levin et al. (37). One strain again formed a clumpy precipitate, which we found to be associated with IPTG induction of our *tolC* vector. We also attempted to optimize the protocol for the method of heat-killing to prevent bacterial growth and to ensure heat-killed cells were intact (determined by phase-contrast microscopy), but we could not detect adsorption above a no-cell control. We then attempted to optimize the Levin et al. (37) protocol using an alternative medium (M9 minimal medium) to see if we could avoid the clumping effects. While this fixed the precipitate issue, we were still unable to detect adsorption rates above the no-cell control level. We tried to further modify the protocol by using Davis minimal medium + 2,000 μg/mL glucose (DM2000). This medium prevented cell precipitate but again did not result in detectable adsorption above the no-cell control level. Suspecting that phage might be “sticking” to the glassware we used, we further attempted to optimize this method by using plastic tubes for the assays. This reduced the overall number of noncell-associated virus decay but again resulted in similar levels between plus-cells and no-cells treatments.

### Phage DNA sequencing.

For clonal phage isolates, DNA was isolated from high-titer phage stocks using the Norgen Phage DNA isolation kit (cat. no. 46800) according to manufacturer’s instructions and sequenced at SeqCenter (seqcenter.com) using Illumina sequencing. Sample libraries were prepared using Nextera-based library preparation (38) using IDT for Illumina 10 bp indices. DNA from clones RB-020 through RB-028 were sequenced on an Illumina NextSeq 550 and Clone ET013 was sequenced on an Illumina NextSeq 2000, producing 2 × 151-bp reads. For mixed populations, DNA was isolated from the full community (bacteria plus phage) revived from frozen mixed communities grown overnight at 37°C. The full-community DNA was isolated using the Qiagen DNeasy blood and tissue kit (cat. no. 69504) and then sequenced at Felix Biotechnology on an Illumina NextSeq 550 platform using Nextera DNA Flex amplicon library preparation. Postsequencing, quality control measures were conducted by the sequencing provider for individual clones: for RB-020 through RB-028, the metrics from the sequencer were used to guarantee that the number of bases with a quality score of Q30 or higher meets or exceeds the total ordered. The data were demultiplexed, and adapters were removed using bcl2fastq (Illumina). For ET013, demultiplexing, quality control, and adapter trimming was performed with bcl-convert (v3.9.3; Illumina).

### Mutation calling.

Mutations were identified by comparing the evolved phage DNA reads to the U136B reference genome (GenBank no. MW598258.1) (11) using *breseq* (26). Clones were analyzed in standard mode with *breseq* v.0.35.4 for ET013 and v.035.5 for all other isolates; these versions vary in minor bug fixes that do not affect calling of the mutation types observed in our isolates. Mixed populations were analyzed with *breseq* v.0.35.5 in polymorphism mode. All other *breseq* parameters were used in their default settings, including those for quality control. Amino acid characteristics were characterized according to the classification system based on charge, polarity, and volume (39). For analysis of our lab stock of phage U136B preexisting mutations, we used our previously published sequencing reads (Sequence Read Archive Accession [SRA] no. SRR13337692).

### Data availability.

All new sequence data are publicly available at SRA BioProject no. PRJNA608759.
