# Supplementary material for: Experimental Evolution of the TolC-Receptor Phage U136B Functionally Identifies a Tail Fiber Protein Involved in Adsorption through Strong Parallel Adaptation
Source: Appl Environ Microbiol. 2023 May 16;89(6):e00079-23. doi: 10.1128/aem.00079-23 (PMC10304864; doi:10.1128/aem.00079-23)
Supplement: Supplemental file 1 — Supplemental material. Download aem.00079-23-s0001.docx, DOCX file, 0.6 MB [file aem.00079-23-s0001.docx]

**Supplementary Material for**

Experimental evolution of the TolC-receptor phage U136B functionally identifies a tail fiber protein involved in adsorption through strong parallel adaptation

Alita R. Burmeister, Eddy Tzintzun-Tapia, Carli Roush, Ivan Mangal, Roxanna Barahman, Robert D. Bjornson, and Paul E. Turner

**SUPPLEMENTARY TABLE**

**Table S1.** Summary of whole-genome sequence data. *Phage* indicates the phage isolate or population described in the main text. All sequence files and metadata (sequencing methods/platform, number of reads, number of bases, etc.) are publicly available on Sequence Read Archive Accession #PRJNA608759. *Genome Coverage* lists the mean fit coverage reported by *breseq* output for phage clones and populations where phage DNA detected, and not applicable (NA) indicates no phage DNA was detected (see main text for description of phage extinctions).

| **Phage** | **Names of Sequences in the Sequence Read Archive BioProject Accession #PRJNA608759** | **Total # of Reads** | **Genome**  **Coverage** | |
| --- | --- | --- | --- | --- |
| RB-020 | Isolated from U136B-E. coli Coevolution Experiment Feb 2019 Pop +2 | 2704219 | 6478.1 | |
| RB-021 | Isolated from U136B-E. coli Coevolution Experiment Feb 2019 Pop +2 | 2558225 | 6162.9 | |
| RB-022 | Isolated from U136B-E. coli Coevolution Experiment Feb 2019 Pop +4 | 4226762 | 9884.4 | |
| RB-023 | Isolated from U136B-E. coli Coevolution Experiment Feb 2019 Pop +4 | 3431671 | 7909.5 | |
| RB-024 | Isolated from U136B-E. coli Coevolution Experiment Feb 2019 Pop +6 | 2395388 | 5642.3 | |
| RB-025 | Isolated from U136B-E. coli Coevolution Experiment Feb 2019 Pop +6 | 3133270 | 7836.6 | |
| RB-026 | Isolated from U136B-E. coli Coevolution Experiment Feb 2019 Pop +6 | 3352846 | 8205.4 | |
| RB-027 | Isolated from U136B-E. coli Coevolution Experiment Feb 2019 Pop +8 | 2529923 | 6240.2 | |
| RB-028 | Isolated from U136B-E. coli Coevolution Experiment Feb 2019 Pop +8 | 2792962 | 7004.9 | |
| ET013 | Pop +9 Isolate #1 from day 10 U136B-E. coli Coevolution Experiment Feb 2019 | 6489729 | 18286.6 |  |
| Pop +1 | Community sample of 2019 E. coli+U136B evolution experiment, Day 10, Pop+1 | 18446631 | NA |  |
| Pop +2 | Community sample of 2019 E. coli+U136B evolution experiment, Day 10, Pop+2 | 13234621 | 5539.6 |  |
| Pop +3 | Community sample of 2019 E. coli+U136B evolution experiment, Day 10, Pop+3 | 12880157 | NA |  |
| Pop +4 | Community sample of 2019 E. coli+U136B evolution experiment, Day 10, Pop+4 | 17316554 | 21964.5 |  |
| Pop +5 | Community sample of 2019 E. coli+U136B evolution experiment, Day 10, Pop+5 | 17669029 | NA |  |
| Pop +6 | Community sample of 2019 E. coli+U136B evolution experiment, Day 10, Pop+6 | 16532841 | 10310.5 |  |
| Pop +7 | Community sample of 2019 E. coli+U136B evolution experiment, Day 10, Pop+7 | 17166464 | NA |  |
| Pop +8 | Community sample of 2019 E. coli+U136B evolution experiment, Day 10, Pop+8 | 20697923 | 257.5 |  |
| Pop +9 | Community sample of 2019 E. coli+U136B evolution experiment, Day 10, Pop+9 | 19645256 | NA |  |
| Pop +10 | Community sample of 2019 E. coli+U136B evolution experiment, Day 10, Pop+10 | 21458311 | NA |  |

**SUPPLEMENTARY FIGURE**

.
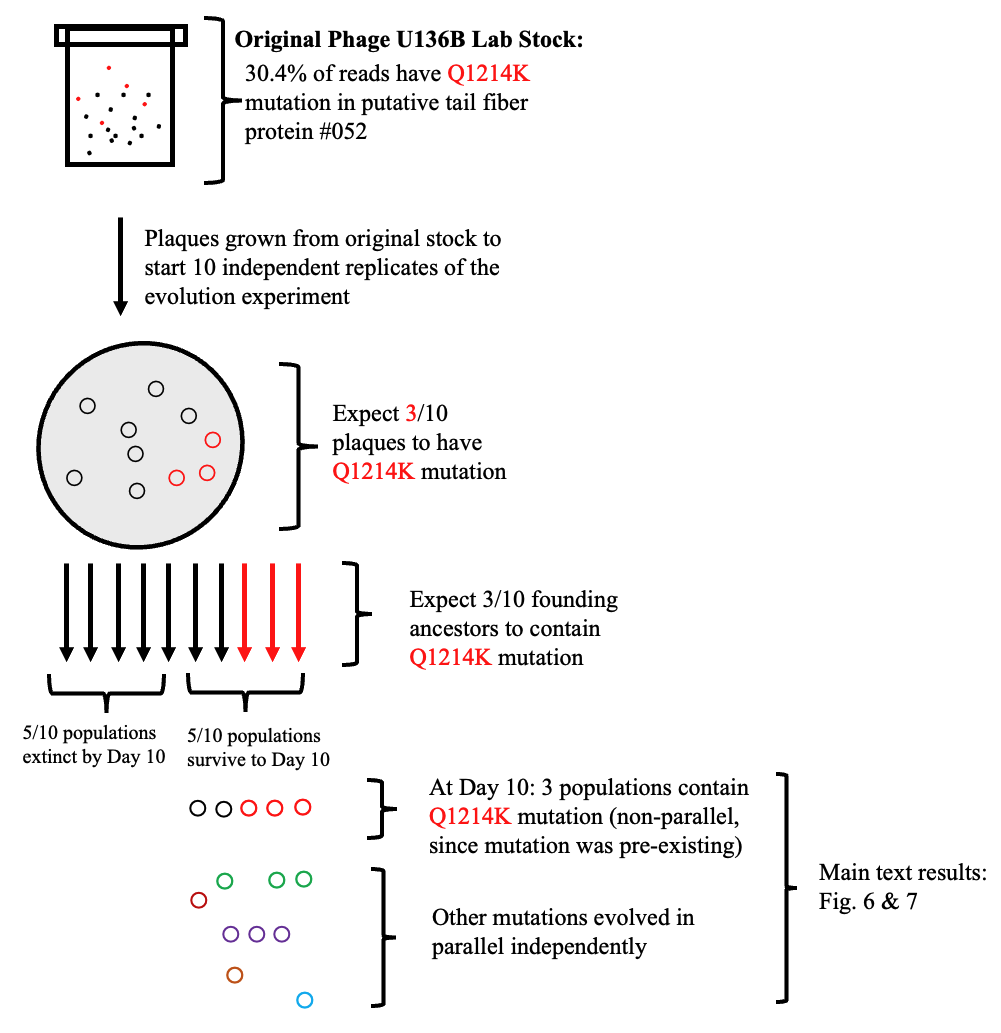


**Figure S1.** Conceptual model of mutation Q1214K in the pre-ancestral phage stock and parallel evolution of other mutations. See main text for details.


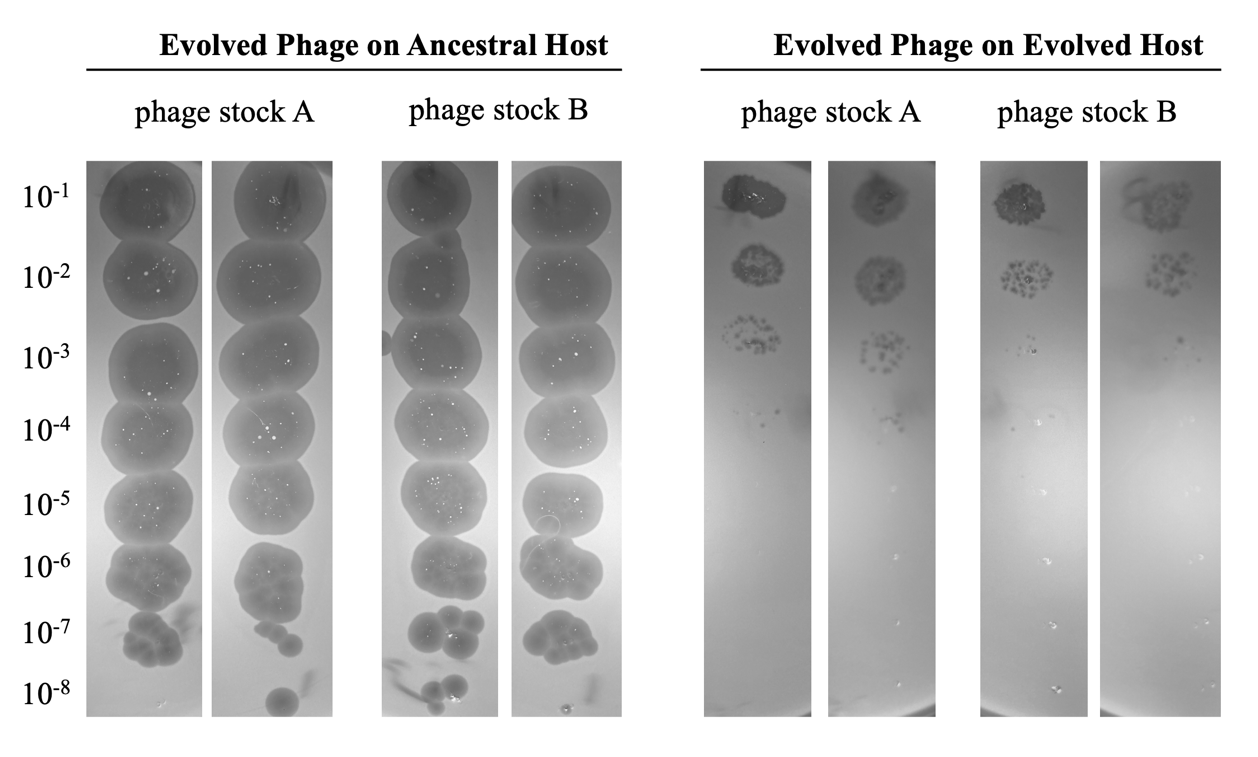


**Figure S2.** Efficiency of plaquing (EOP) of the evolved phage isolate (RB-020) on both its ancestral (BW25113) and evolved (AB279) hosts. The evolved phage has a lower EOP on its evolved host than on the ancestral host (<10^-4^), but this effect is not representative of its evolutionary fitness on the evolved host. EOPs (and plaquing character in general) do not well-represent key phage fitness components, such as adsorption rate (main text and main Fig. 5B). Each assay was conducted with two replicate phage stocks (A and B), each with two technical replicates (individual 1:10 dilutions series with 2 µl plated at each spot).
